# Sense and antisense RNA are not toxic in *Drosophila* models of *C9orf72*-associated ALS/FTD

**DOI:** 10.1007/s00401-017-1798-3

**Published:** 2018-01-29

**Authors:** Thomas G. Moens, Sarah Mizielinska, Teresa Niccoli, Jamie S. Mitchell, Annora Thoeng, Charlotte E. Ridler, Sebastian Grönke, Jacqueline Esser, Amanda Heslegrave, Henrik Zetterberg, Linda Partridge, Adrian M. Isaacs

**Affiliations:** 1 Department of Neurodegenerative Disease, UCL Institute of Neurology, London, WC1N 3BG UK; 2 Department of Genetics, Evolution and Environment, Institute of Healthy Ageing, University College London, London, WC1E 6BT UK; 3 Department of Basic and Clinical Neuroscience, Maurice Wohl Clinical Neuroscience Institute, Institute of Psychiatry, Psychology and Neuroscience, King’s College London, London, SE5 9RT UK; 4 UK Dementia Research Institute at King’s College London, Maurice Wohl Clinical Neuroscience Institute, Institute of Psychiatry, Psychology and Neuroscience, London, SE5 9RT UK; 5 Max Planck Institute for Biology of Ageing, 50931 Cologne, Germany; 6 Department of Molecular Neuroscience, UCL Institute of Neurology, London, WC1N 1PJ UK; 7 Clinical Neurochemistry Laboratory, Department of Psychiatry and Neurochemistry, Institute of Neuroscience and Physiology, The Sahlgrenska Academy, University of Gothenburg, Gothenburg, Sweden; 8 UK Dementia Research Institute at UCL, UCL Institute of Neurology, London, WC1N 3BG UK

**Keywords:** *C9orf72*, *Drosophila*, ALS, FTD, Repeat expansion

## Abstract

A GGGGCC hexanucleotide repeat expansion in the *C9orf72* gene is the most common genetic cause of amyotrophic lateral sclerosis and frontotemporal dementia. Neurodegeneration may occur via transcription of the repeats into inherently toxic repetitive sense and antisense RNA species, or via repeat-associated non-ATG initiated translation (RANT) of sense and antisense RNA into toxic dipeptide repeat proteins. We have previously demonstrated that regular interspersion of repeat RNA with stop codons prevents RANT (RNA-only models), allowing us to study the role of repeat RNA in isolation. Here we have created novel RNA-only *Drosophila* models, including the first models of antisense repeat toxicity, and flies expressing extremely large repeats, within the range observed in patients. We generated flies expressing ~ 100 repeat sense or antisense RNA either as part of a processed polyadenylated transcript or intronic sequence. We additionally created *Drosophila* expressing > 1000 RNA-only repeats in the sense direction. When expressed in adult *Drosophila* neurons polyadenylated repeat RNA is largely cytoplasmic in localisation, whilst intronic repeat RNA forms intranuclear RNA foci, as does > 1000 repeat RNA, thus allowing us to investigate both nuclear and cytoplasmic RNA toxicity. We confirmed that these RNA foci are capable of sequestering endogenous *Drosophila* RNA-binding proteins, and that the production of dipeptide proteins (poly-glycine–proline, and poly-glycine–arginine) is suppressed in our models. We find that neither cytoplasmic nor nuclear sense or antisense RNA are toxic when expressed in adult *Drosophila* neurons, suggesting they have a limited role in disease pathogenesis.

**Electronic supplementary material:**

The online version of this article (10.1007/s00401-017-1798-3) contains supplementary material, which is available to authorized users.

## Introduction

Amyotrophic lateral sclerosis (ALS) and frontotemporal dementia (FTD) are two adult onset, neurodegenerative diseases, leading to loss of the upper and lower motor neurons, or frontal and temporal lobe cortical neurons, respectively. In recent years, it has become apparent that these diseases represent extremes of a clinical and pathological spectrum of disorders, since genetic evidence shows that either or both of these clinical manifestations can be caused by mutations in the same set of genes [33].

A (GGGGCC)_*n*_ hexanucleotide expansion within the first intron of the *C9orf72* gene is the most common genetic cause of both ALS and FTD, causing up to 40% of familial ALS and 25% of familial FTD [16, 35, 45]. Unaffected individuals typically carry 2–10 repeats, whilst patients harbour hundreds to thousands [16, 45]. Toxicity has been proposed to result either from a loss of function of the *C9orf72* gene, or a toxic gain of function [46]. Neuron-specific, or whole genome knockout of the mouse ortholog of *C9orf72* does not lead to a neurodegenerative phenotype, suggesting that toxicity primarily arises through a gain of function mechanism [3, 8, 26, 29, 31, 51, 52]. Two such mechanisms have been proposed: RNA toxicity, or toxicity arising due to proteins derived from the repeat RNA [39].

The hexanucleotide repeat can be transcribed in both sense and antisense directions, resulting in sense and antisense RNA foci, typically nuclear aggregates of RNA. These have been proposed to exert toxicity by sequestering RNA-binding proteins [16, 20, 38, 45, 59]. Additionally, sense and antisense RNA can be translated in all reading frames, via repeat-associated non-ATG initiated translation (RANT), producing five dipeptide proteins (DPRs): poly-GR, poly-GA, and poly-GP from the sense stand, and poly-PR, poly-AP, and poly-GP from the antisense [2, 40, 59].

We and others have previously demonstrated that expression of hexanucleotide RNA is extremely toxic to adult *Drosophila* neurons, resulting in dramatically shortened lifespan [19, 37, 56]. However, insertion of regularly interspersed stop codons (“RNA-only” constructs) within the hexanucleotide sequence rescues toxicity, suggesting that it is primarily mediated by DPRs [37]. Multiple groups have demonstrated that among the DPRs, poly-GR, and poly-PR are the most toxic, with poly-GA exerting toxicity in some model systems [39].

Despite these advances, the potential role of RNA toxicity in *C9orf72* mutation-associated ALS/FTD is not fully resolved. Recent work has suggested that expression of sense RNA is toxic to rat primary neurons [54] and that cytoplasmic rather than nuclear RNA may drive pathology [9]. The role of antisense RNA in isolation has not yet been assessed.

RNA-only constructs provide the ideal tool to assess a role of *C9orf72* repeat RNA without the confounding effect of RANT DPRs. Here we examine whether genomic context or repeat length can cause RNA-only (RO) repeats to be toxic to *Drosophila* neurons. We find that expressing RO repeats as part of an intron potentiates sense and antisense intranuclear RNA foci formation, as does expressing extremely long repeats within the range observed in patients (> 1000 repeats). However, we find no evidence of toxicity arising due to sense or antisense repeat RNA, despite observing sequestration of RNA-binding proteins by RNA foci.

## Materials and methods

### Generation of DNA constructs

Recursive directional ligation was used to generate 108RO and ~ 1152RO constructs in the pBluescript vector as previously described [37]. The repeat sequence was subsequently subcloned into the pUAST attB *Drosophila* transgenesis vector to generate sense polyA constructs. For antisense polyA constructs, the 108RO sequence was cloned in reverse. For the sense intronic construct: the pUAST attB vector was modified to remove *Xba*I and *Bam*HI restriction sites. The pGint vector [7] (Addgene Plasmid #24217) was digested with *Bgl*II and *Not*I to liberate the eGFP coding sequence, and this was subcloned into a modified pUAST attB vector (pUAST attB eGFP). Following this, repeats were subcloned from the sense-polyA vector into the intronic region using the *Bam*HI and *Xba*I restriction sites within the eGFP intronic sequence. To generate antisense intronic constructs: the origin of replication of the pUAST attB eGFP vector was reversed, the original *Eco*RI site was removed from upstream of the eGFP sequence, and an *Eco*RI cut site was introduced into the intronic region of the eGFP sequence, the repeats were cloned in antisense orientation from the sense-polyA vector using *Eco*RI and *Xba*I.

Plasmids were propagated and purified in the manner described previously [37]. Repeat size was confirmed using DNA digestion (~ 1152RO construct) and/or sequencing with dGTP (Source Bioscience). Due to minor repeat contraction events, the final repeat numbers in each plasmid are: 108 repeats (sense polyA and antisense intronic constructs), 107 repeats (antisense polyA construct) and 106 (sense intronic construct). Sequences are available on request.

### Generation of transgenic *Drosophila*

Constructs were inserted using phiC31-integrase-mediated, site-directed insertion at the attP40 locus [36]. Constructs were injected into y1, M{vas-int.B}ZH-2A w*; P{CaryP}attP40 embryos and the phiC31 integrase was removed by crossing transgenic males to females for two successive generations before use in phenotyping experiments.

### *Drosophila* stocks and maintenance

*Drosophila* stocks were maintained on SYA food (15 g/L agar, 50 g/L sugar, 100 g/L autolysed yeast, 30 mL/L nipagin (10% in ethanol) and 3 mL/L propionic acid) at 25 °C in a 12-h light–dark cycle with constant humidity. For RU486-induced experiments, food was supplemented with 200 μM RU486 (mifepristone).

The elavGS stock was generously provided by Herve Tricoire (Paris Diderot University) [43]. The GMR-Gal4 line was obtained from the Bloomington *Drosophila* Stock Centre. The Glorund RNAi line was obtained from the Vienna Drosophila Resource Center (GD12082, v27752). All experiments were performed on mated females, unless otherwise stated.

### Lifespan assays

Flies were raised at standard density in 200-mL bottles. After eclosion, flies were allowed to mate for 48 h. 135–150 flies of the indicated genotype were split into groups of 15 and housed in vials containing SYA medium with or without RU486. Deaths were scored, and flies tipped onto fresh food three times a week. Data are presented as cumulative survival curves. All lifespans were performed at 25 °C.

### Negative geotaxis assays

Negative geotaxis assays were performed as described previously, either using technical replicates of ~ 75 flies in a glass-walled chamber [41], or 3 replicates of 15 flies in plastic pipettes [49] (Fig. 2). Performance indexes were calculated as described previously [49].

### Fluorescence in situ hybridization (FISH)

Adult females of the indicated genotype were allowed to feed on RU486 containing food for 7 days. Brains were dissected and FISH performed as described elsewhere [53]. The following probes were used: Cy3-labelled (GGCCCC)_4_ 2′-O-methyl RNA probe (Integrated DNA Technologies) [38], and 5′ TYE563-labelled (GGGGCC)_3_ probe (Exiqon). Following the FISH protocol, brains were mounted in Vectashield mounting medium with DAPI (Vectorlabs). Images were taken using a Zeiss LSM 700 confocal microscope, using the 63× lens and the same settings within each experiment. For foci quantification, images were taken without prior visualization of the Cy3 channel to avoid experimenter induced bias. Numbers of nuclei and foci containing nuclei were scored with experimenter blinded to genotype.

### FISH immunofluorescence

Following the FISH protocol, brains were blocked for 1 h in blocking solution [PBS with 0.3% Triton X-100 and 10% BSA (Sigma)], before being incubated overnight at 4°C with anti-Glorund primary antibody (5B7, Developmental Studies Hybridoma Bank) at a concentration of 1/100 in blocking solution. The following day the tissue was washed three times in PBST before being incubated in Alexa-488-conjugated goat anti-mouse secondary antibody (A11001, Thermo Fisher) at 1/1000 in block at room temperature for 1 h. Following three further washes, the brains were mounted as described above. Images were taken using a Zeiss LSM 700 confocal microscope, using the same settings. Foci overlap with Glorund puncta was quantified automatically using Volocity analysis software (Perkin Elmer).

### Dipeptide immunoassays

Antibodies were produced by immunising rabbits with either (GP)_7_, or (GR)_7_ followed by affinity purification and a proportion biotinylated (Eurogentec) [48]. Adult flies of the indicated genotypes were fed food containing 200 µM RU486 or control food for 7 days. Flies were frozen in liquid nitrogen and heads removed. 7–10 heads per replicate were homogenised in ice-cold RIPA buffer (Sigma) with protease inhibitors (Roche cOmplete mini EDTA-free). Lysis was allowed to proceed on ice for 10 min, before lysates were centrifuged at 21,000×*g* for 20 min at 4 °C. Supernatant was collected in fresh tubes. The concentration of protein was determined using the DC protein assay (BioRad). Concentration of lysates were made equivalent by the addition of further RIPA buffer with protease inhibitors. The total amount of protein loaded per well in each assay was 24 μg for poly-GR and 14 μg for poly-GP.

Immunoassays were performed using 96-well SECTOR plates (MSD, Rockville, Maryland) as described previously [48, 50]. Detection was performed using an MSD sector imager. Specificity was confirmed with a peptide cross reactivity assay using (GP)_7_, (GR)_7_, (GA)_7_, (PR)_7_, and (AP)_7_ synthetic peptides (Biogenes) at a concentration of 100 ng/mL. For *Drosophila* assays lysates from wild-type flies (w1118) were run on each plate, and the resultant values subtracted from all samples to correct for background. A four-parameter logistic regression curve was fit to the values obtained from a standard curve of peptide calibrators using graph pad prism, and concentrations were interpolated.

### Statistics

Lifespan data were compared using log rank test. For negative geotaxis analysis, counts of flies at each height were compared using ordinal logistic regression. For percentage foci positive nuclei data, a linear model with genotype as the explanatory variable was fitted. Comparisons were made using a priori orthogonal contrasts.

## Results

### Generation of transgenic *Drosophila* expressing sense RNA-only repeats either within an intron or as a polyadenylated transcript

**Fig. 1:**
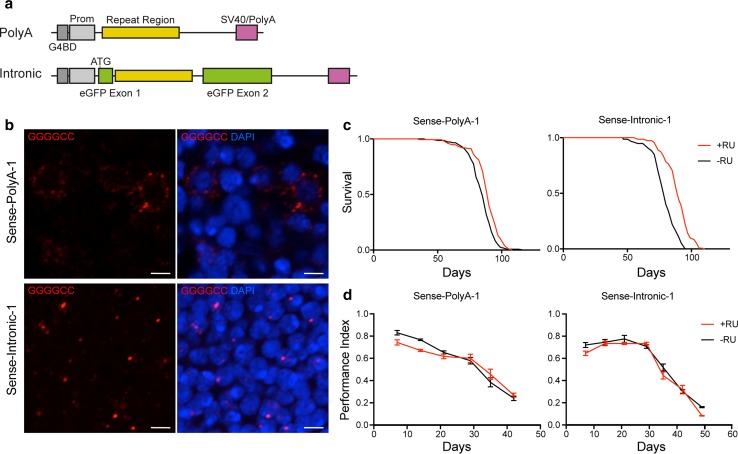
Expression of *C9orf72* sense RNA-only repeats is not strongly toxic to *Drosophila* neurons. **a** Diagram of the constructs generated. RO repeats were either cloned directly into the vector, forming part of a polyadenylated transcript (PolyA) or cloned into an intron within the eGFP coding sequence (Intronic). *5× Gal4 DNA binding domain (G4BD), heat-shock promoter (Prom), late SV40 termination and polyadenylation sequence (SV40/PolyA).* **b** Fluorescence in situ hybridisation was performed to assess foci formation (GGGGCC, red). Expression was induced in adult *Drosophila* neurons using the elavGS driver, leading to the formation of largely cytoplasmic RNA puncta in Sense-PolyA-1 flies, and intranuclear RNA foci in Sense-Intronic-1 flies. Scale bar 2.5 μm. **c** Analysis of lifespan of Sense-PolyA-1 and Sense-Intronic-1 flies fed RU486 to induce expression (+RU) or controls where expression was not induced (−RU). In both cases a significant lifespan extension was observed upon transgene expression (Sense-PolyA-1, median lifespan −RU = 83.5 days +RU = 89 days, *P* = 6.91E−5, log rank test; Sense-Intronic-1, median lifespan −RU = 79 days +RU = 89 days, *P* = 9.76E−19, log rank test). **d** Negative geotaxis assays performed on Sense-PolyA-1 and Sense-Intronic-1 flies expressing the transgene (+RU) and controls (−RU). A slight reduction in climbing ability is observed in Sense-PolyA-1 flies (ordinal logistic regression, interaction of RU status and time *P* = 0.00025), whilst no significant effect is seen in Sense-Intronic-1 flies *P* = 0.47). Error bars are ± SEM. Genotypes: w; UAS-Sense-PolyA-1/+; elavGS/+ (Sense-PolyA-1), w; UAS-Sense-Intronic-1/+; elavGS/+ (Sense-Intronic-1)

We have previously created *Drosophila* capable of inducible expression of 108 sense RO repeats as part of a capped and polyadenylated (polyA) transcript [37]. However, the genomic location of the repeat region in humans is either within the first intron of the *C9orf72* gene or within the promoter, depending on the splice variant [16, 45]. We, therefore, created a *Drosophila* model capable of expressing 106 RO hexanucleotide repeats from within a strongly, constitutively, spliced artificial intron introduced into an eGFP coding sequence (Fig. 1a) [7]. Constructs were integrated at the same genomic site to ensure equivalent expression levels. Because plasmids containing repetitive DNA elements are unstable in bacteria, and repeat length tracts can be unstable in *Drosophila* [28], we derived multiple independent transgenic lines and screened for repeat expansions of the correct size using Southern blotting (Online Resource Fig. 1) and selected two lines capable of expressing ~ 100 RO sense repeats either as part of a processed RNA transcript (Sense-PolyA-1 and Sense-PolyA-2) or from an intron (Sense-Intronic-1 and Sense-Intronic-2). We additionally confirmed expression of eGFP in intronic flies, indicating that the intronic repeat region is efficiently spliced out of the mature mRNA transcript (Online Resource Fig. 2).

### Sense polyA repeat RNA accumulates in the cytoplasm and intronic repeat RNA accumulates in the nucleus

**Fig. 2:**
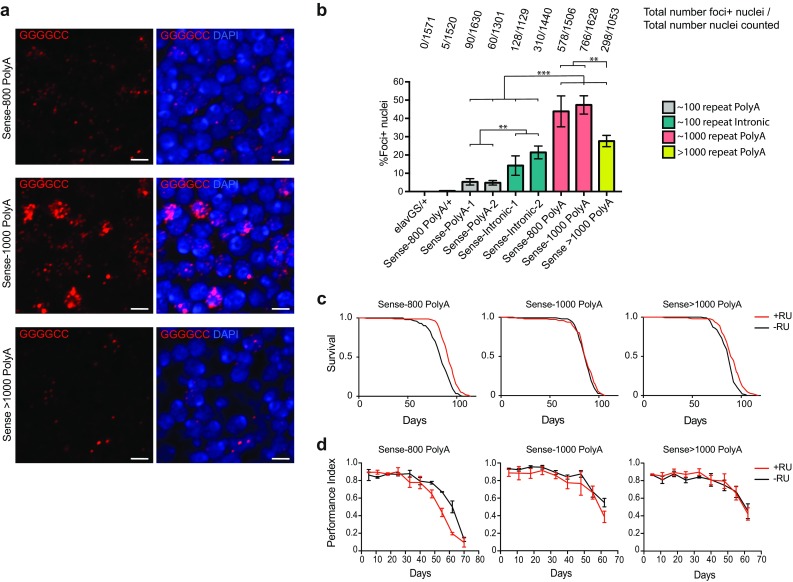
Expression of ~ 1000 RNA-only repeats or greater causes the production of large numbers of RNA foci but does not induce strong toxicity when expressed in adult *Drosophila* neurons. **a** Fluorescence in situ hybridisation against sense RNA foci reveals abundant RNA foci are present in all lines expressing ~ 1000 RO repeats (Sense-800 PolyA and Sense-1000 PolyA) and Sense > 1000 RO repeats (Sense > 1000 PolyA). Scale bar 2.5 μm. **b** Quantification of the % of foci containing nuclei (%foci+ nuclei) within each line. No foci+ nuclei are detected in driver alone (elavGS/+) and very few observed in transgene without the driver (Sense-800 PolyA/+) lines. A linear model was fitted to the data (effect of genotype *P* < 0.0001), and comparisons between groups made using orthogonal contrasts (all contrasts shown in Online Resource Table 1). A significantly higher proportion of nuclei were foci+ in ~ 100 repeat Sense-Intronic flies vs. ~ 100 repeat PolyA flies (**P = 0.0071). A significantly higher proportion of foci+ nuclei were observed in 800 to > 1000 repeat-expressing flies compared to ~ 100 repeat-expressing flies (***P < 0.0001). Fewer foci were observed in > 1000-repeat-expressing flies vs. 800–1000 repeat (**P = 0.0039). 3–4 brains per genotype were examined. Bars are mean ± SEM. **c** Lifespans of flies expressing long sense constructs (+RU) vs. controls (−RU) using the elavGS driver. Significant lifespan extensions are observed in Sense-800 PolyA (median lifespan −RU = 83.5 days +RU = 90.5 days, *P* = 4.07E−10 log rank test) and Sense > 1000 PolyA-expressing flies (median lifespan −RU = 86.5 days +RU = 90.5 days, *P* = 4.21E−06 log rank test). Lifespan of Sense-1000 PolyA-expressing flies was not significantly different (median lifespan −RU = 86.5, +RU = 86.5, *P* = 0.75, log rank test). **d** Negative geotaxis assays performed on flies expressing the transgene (+RU) and controls (−RU) using the elavGS driver. In Sense-800 PolyA flies, a slight reduction in climbing ability is observed with age vs. controls (ordinal logistic regression, interaction of RU status and time *P* = 0.0281); however, no significant differences were observed in Sense-1000 PolyA (*P* = 0.328) or Sense > 1000 PolyA (*P* = 0.231)-expressing flies. Error bars are ± SEM. Genotypes: w; +; elavGS/+ (elavGS/+), w; UAS-Sense-800 PolyA/+; + (Sense-800 PolyA/+), w; UAS-Sense-PolyA-1/+; elavGS/+ (Sense-PolyA-1), w; UAS-Sense-Intronic-1/+; elavGS/+ (Sense-Intronic-1), w; UAS-Sense-800 PolyA/+; elavGS/+ (Sense-800 PolyA), w; UAS-Sense-1000 PolyA/+; elavGS/+ (Sense-1000 PolyA), w; UAS-Sense > 1000 PolyA/+; elavGS/+ (Sense > 1000 PolyA)

To test whether the genomic context of the repeat alters the formation of RNA foci in our models we performed RNA fluorescent in situ hybridisation (FISH) in adult *Drosophila* neurons. Sense polyA or sense intronic RO repeats were expressed in adult neurons using the pan-neuronal, inducible, Elav gene-switch (elavGS) driver, with expression induced after eclosion. In both types of fly, foci were observed in adult neurons (Fig. 1b) and not in driver-only controls (Fig. 2b, Online Resource Fig. 3). In sense polyA RO repeat-expressing flies, we observed a primarily cytoplasmic RNA signal in adult neurons, with occasional (5–6% of cells) intranuclear puncta (Figs. 1b, 2b). However, in sense intronic RO repeat-expressing flies we observed a shift in RNA localisation to predominantly nuclear RNA foci, present in 11–22% of neurons (Fig. 2b). The differential localisation of RNA in these lines allows investigation of both cytoplasmic and nuclear *C9orf72* repeat RNA toxicity.

### Sense cytoplasmic RNA and nuclear RNA foci do not cause typical neurodegenerative phenotypes

To determine whether *C9orf72* sense RNA can lead to neurodegenerative phenotypes, we assessed the effect of adult pan-neuronal expression of sense polyA and sense intronic RO repeats on the survival of *Drosophila*. We observed that expression of sense polyA or sense intronic repeats did not reduce adult survival compared to controls where no expression was induced; in fact, a significant extension of lifespan was observed (Fig. 1c, Online Resource Fig. 4a, c). This lifespan extension did not occur in flies carrying the driver alone, or expressing a non-toxic DPR protein inserted in the same locus (Online resource Fig. 5). As an alternative metric of neuronal health, we assessed the climbing ability of adult flies using negative geotaxis assays. For sense polyA expressing flies, we observed a slight reduction in climbing ability with age across both replicates compared to controls, but saw no consistent reduction in climbing ability with age in sense intronic RO repeat-expressing flies (Fig. 1d, Online Resource Fig. 6a, c). We have previously shown that expression of pure (non RNA-only) repeats during development at high temperatures, a mildly stressful condition, led to a very strong eye phenotype [37]. To analyse whether the RO repeats would also show a phenotype under these conditions we expressed the constructs in the developing *Drosophila* compound eye using the GMR-Gal4 driver, assessing both roughness of the adult eye and the percentage of pupae that eclosed (Online Resource Fig. 7). No RO lines, polyA or intronic, showed an eclosion defect. A slight rough eye phenotype was observed in female (but not male) flies expressing polyA repeats, potentially due to the production of small amount of DPRs in these flies (see “Discussion”). Intronic repeat expressing flies did not demonstrate a rough eye phenotype or eclosion defect, consistent with a lack of toxicity of nuclear RNA foci.

### Expression of > 1000 sense RNA-only repeats generates many RNA foci

*C9orf72* ALS/FTD patients typically carry thousands of repeats [4, 5]. We, therefore, consider that we might not yet have achieved a repeat length sufficient to induce RNA toxicity. To test this hypothesis, we generated *Drosophila* capable of expressing ~ 1000 RNA-only repeats as part of a polyadenylated transcript. Using Southern blotting, we identified two lines: Sense-800 PolyA and Sense-1000 PolyA, which harboured ~ 800 and ~ 1000 repeats, respectively (Online Resource Fig. 8). In addition, we found lines carrying expansions larger than expected, consistent with de novo expansion events. We, therefore, selected one of these lines Sense-> 1000 PolyA (approximately 2000–5000 repeats) for further characterisation (Online Resource Fig. 8).

When we expressed these constructs in adult neurons, we observed abundant nuclear and cytoplasmic RNA foci in up to 47% of cells (Fig. 2a, b). Unexpectedly we observed a lower number of foci in Sense > 1000 PolyA flies compared to Sense-800 PolyA and Sense-1000 PolyA flies. In addition to the driver control, we did not observe an appreciable number of foci (< 0.5% of nuclei) in flies carrying (but not expressing) the repeat transgene (Fig. 2b, Online Resource Fig. 3), indicating that signal is not caused by the probe interacting non-specifically with other cellular components, including genomic DNA.

### Expression of > 1000 sense RNA-only repeats does not cause typical neurodegenerative phenotypes

Despite the presence of abundant RNA foci, Sense-800 PolyA, Sense-1000 PolyA and Sense > 1000 PolyA flies did not show a reduction in lifespan in females (Fig. 2c). We additionally assessed lifespan in male flies, and observed a significant but extremely modest reduction in lifespan with repeat expression (Online Resource Fig. 9). Whilst we observed a small decline in climbing ability of Sense-800 PolyA flies at late ages this was not consistently replicated in either Sense-1000 PolyA flies or Sense > 1000 PolyA flies (Fig. 2d). We additionally failed to observe a rough eye phenotype or eclosion defect when constructs were expressed using the GMR-Gal4 driver (Online Resource Fig. 7).

To ensure that the lack of typical neurodegenerative phenotypes was not caused by repeat length instability, we performed Southern blotting on the flies used in the lifespan and negative geotaxis assays, and observed conservation of repeat length (Online Resource Fig. 8b).

### Antisense polyA RNA repeats accumulate in the cytoplasm and intronic antisense RNA repeats accumulate in the nucleus

The potential role of antisense *C9orf72* repeat RNA in neurodegeneration has thus far not been investigated in *Drosophila*. Because the stop codons present in the RO constructs are also able to block translation in the antisense direction, we created flies capable of expressing ~ 100 antisense RO repeats by reversing the orientation of the repeat construct. We reasoned that intronic localisation would enhance nuclear antisense RNA foci formation as found for sense repeats; therefore, we created two sets of constructs, polyadenylated and intronic. We derived multiple independent transgenic lines and screened for repeat expansions of the correct size using Southern blotting (Online Resource Fig. 10), deriving two lines capable of expressing ~ 100 RO sense repeats either as part of a processed polyadenylated RNA transcript (AS-PolyA-1 and AS-PolyA-2) or from an intron (AS-Intronic-1 and AS-Intronic-2). eGFP expression was confirmed in intronic flies demonstrating correct splicing (Online Resource Fig. 2). Due to repeat instability, we were unable to create DNA constructs of > 108 antisense RO repeats.

**Fig. 3:**
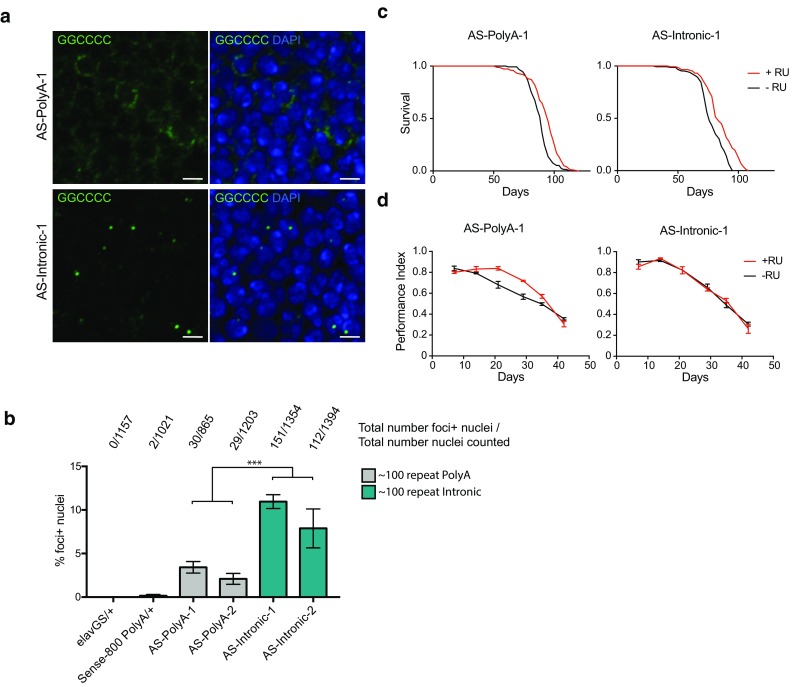
Antisense RNA forms foci, but does not induce strong toxicity when expressed in *Drosophila* neurons. **a** Fluorescence in situ hybridisation was performed to assess antisense foci formation (GGCCCC, green). Expression was induced in adult *Drosophila* neurons using the elavGS driver, leading to largely cytoplasmic RNA signal in AS-PolyA-1 flies, and predominantly intranuclear RNA foci in AS-Intronic-1 flies. Scale bar 2.5 μm. **b** Quantification of the % of foci containing nuclei (%foci+ nuclei) within each line. No foci+ nuclei are detected in driver alone (elavGS/+) and very few observed in transgene alone (Sense-800 PolyA-1/+) lines. A linear model was fitted to the data (effect of genotype *P* < 0.0001), and comparisons between groups made using orthogonal contrasts (all contrasts shown in Online Resource Table 2). A significantly higher proportion of nuclei were foci+ in AS-Intronic flies vs. AS-PolyA flies (***P < 0.0001). 2–4 brains per genotype were examined. Bars are mean ± SEM. **c** Lifespans of flies expressing antisense constructs (+RU) vs. controls (−RU) using the elavGS driver. A significant extension of lifespan is observed in AS-PolyA-1 (median lifespan −RU = 89 days +RU = 93.5 days, *P* = 5.20E−8 log rank test) or AS-Intronic-1 flies (median lifespan −RU = 75.0 days +RU = 82.5 days, *P* = 4.92E−8 log rank test). **d** Negative geotaxis assays performed on AS-PolyA-1 and AS-Intronic-1 flies expressing the transgene (+RU) and controls (−RU) using the elavGS driver. In AS-PolyA-1 flies, no significant difference in climbing ability is observed with age vs. controls (ordinal logistic regression, interaction of RU status and time *P* = 0.988), or AS-Intronic-1-expressing flies (*P* = 0.439). Error bars are ± SEM. Genotypes: w; +; elavGS/+ (elavGS/+), w; UAS-Sense-800 PolyA/+; + (Sense-800 PolyA/+), UAS-AS-polyA-1/+; elavGS/+ (AS-PolyA-1), UAS-AS-Intronic-1/+; elavGS/+ (AS-Intronic-1)

We assessed antisense RNA foci formation in adult *Drosophila* neurons using FISH. As in the sense flies, in antisense polyA expressing flies, we observed frequent cytoplasmic RNA signals in adult neurons, with occasional intranuclear puncta (2–3% of cells, Fig. 3a). As anticipated, in antisense intronic RO repeat-expressing flies we observed a shift in RNA localisation to predominantly nuclear RNA foci, with foci present in approximately 8–11% of cells (Fig. 3b). Therefore, these novel lines allow investigation of both cytoplasmic and nuclear antisense *C9orf72* repeat RNA toxicity.

### Expression of antisense RNA-only *C9orf72* repeats does not cause typical neurodegenerative phenotypes

Flies expressing antisense polyA or intronic RO repeats did not show a reduced lifespan (Fig. 3c, Online resource Fig. 4b, d) or consistent effects on climbing ability (Fig. 3d, Online Resource Fig. 6b, d). In addition, no rough eye phenotype or eclosion defect was observed when antisense constructs were expressed during development using the GMR-Gal4 driver (Online Resources Fig. 7).

### RNA-only repeat RNA foci sequester RNA-binding proteins

**Fig. 4:**
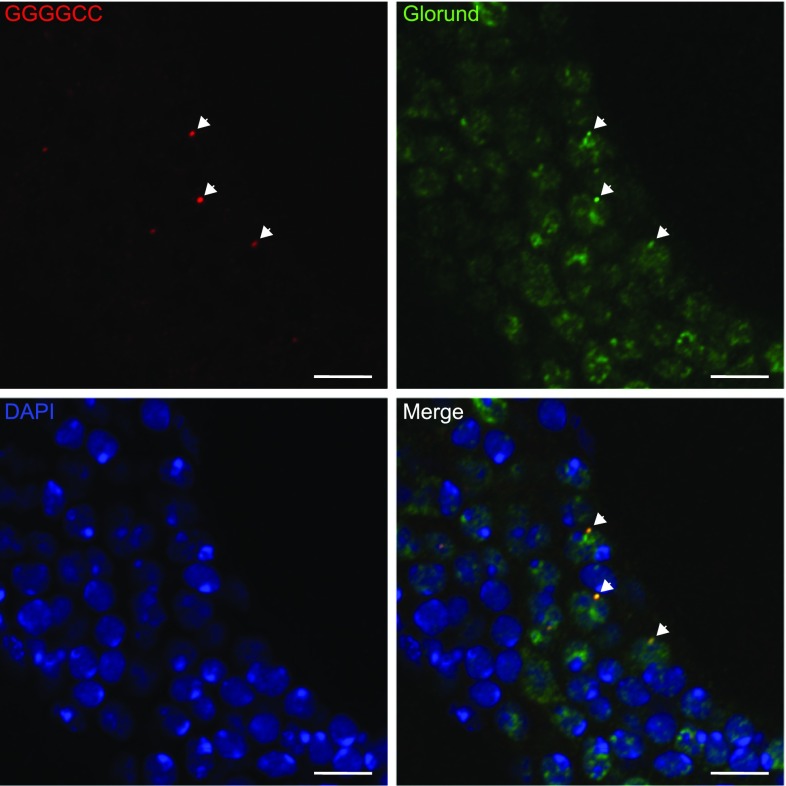
Glorund colocalises with sense RNA foci in adult *Drosophila* neurons. Fluorescence in situ hybridisation against sense foci (GGGGCC, red) was coupled with immunofluorescence against glorund (green). Bright puncta of glorund staining were found to colocalise with sense RNA foci (examples of colocalising puncta shown by white arrowheads). An average of 14.8% of sense RNA foci was found to colocalise with glorund puncta (± 4.26% SEM, based on 167 foci scored across 3 separate brains). Scale bar 5 μm. Genotype: w; UAS-Sense-800 PolyA/+; elavGS/+

Sequestration of RNA-binding proteins is thought to be a critical mediator of RNA toxicity [55]. To verify that the RNA foci formed by RO repeats sequester RNA-binding proteins we performed FISH coupled with immunofluorescence to determine whether the foci present in Sense-800 PolyA-expressing flies could sequester Glorund (Glo), the *Drosophila* ortholog of hnRNP H, which has been identified in multiple independent studies as a *C9orf72* sense RNA foci interacting protein [12, 13, 25, 32]. We found puncta of Glo colocalised with an average of 15% of foci (Fig. 4), showing that foci formed by RO repeats are capable of sequestering endogenous *Drosophila* proteins. Therefore, RO repeat RNA foci recapitulate this key property of RNA foci observed in patient material, without causing overt toxicity.

To examine whether loss of function of Glo alone is sufficient to induce a neurodegenerative phenotype, we knocked down Glo in developing photoreceptor neurons using GMR-Gal4. We observed a significant knockdown when expressing Glo RNAi at 29 °C (Online Resource Fig. 11a), we failed to observe either a rough eye phenotype, or eclosion defect compared to controls (Online Resource Fig. 11b–e). This suggests that even if the RNA foci were sequestering large amounts of Glo, this would be unlikely to translate into a toxic phenotype.

### Production of dipeptide proteins is suppressed from RNA-only repeats

To quantitatively assess whether production of DPRs is prevented in our models, we developed in-house Meso Scale Discovery (MSD) immunoassays, using antibodies specific for poly-GP or poly-GR [57] (Online Resource Fig. 12).

**Fig. 5:**
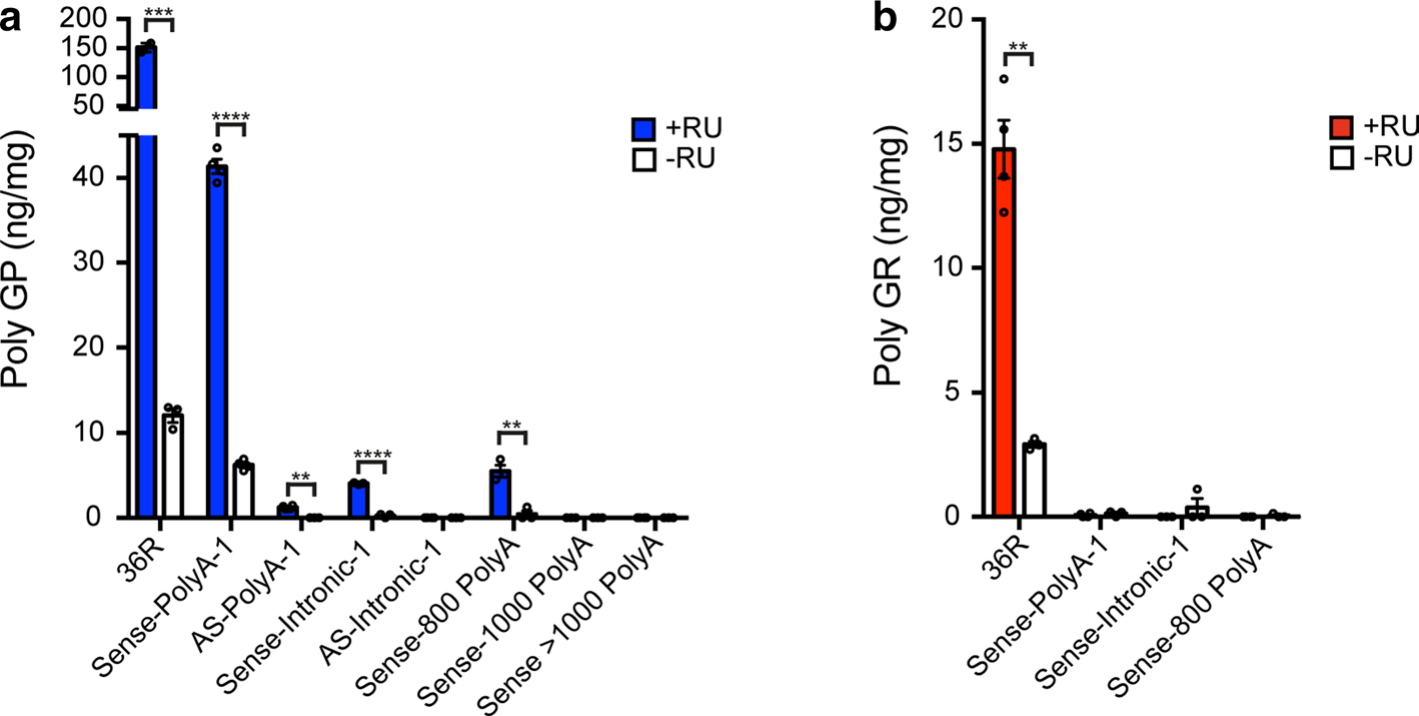
RANT is suppressed in RNA-only flies. **a** Assessment of poly-GP expression. Heads of flies induced on RU486 for 7 days (+RU) and controls (−RU) were lysed and dipeptide protein concentration measured by immunoassay. Poly-GP is detectable in significantly higher abundance in 36R-expressing flies compared to non-induced controls (***P = 0.0002, two-tailed *t* test, +RU *n* = 2, −RU *n* = 3). Additionally a significantly higher level of poly-GP was observed in lines Sense-PolyA-1 (****P < 0.0001, two-tailed *t* test, +RU *n* = 4, −RU *n* = 4), AS-PolyA-1 (**P = 0.0017, Welch’s two-tailed *t* test, +RU *n* = 4, −RU *n* = 3), Sense-Intronic-1 (****P < 0.0001, two-tailed *t* test, both conditions *n* = 3), and Sense-800 PolyA (**P = 0.0036, two-tailed *t* test, both conditions *n* = 3). No differences in poly-GP levels were observed in lines expressing AS-Intronic-1, Sense-1000 PolyA and Sense > 1000 PolyA vs. controls (Sense > 1000 PolyA and AS-Intronic-1 +RU *n* = 4, *n* = 3 for other conditions). **b** Assessment of poly-GR expression. Poly-GR expression was assessed by immunoassay. Poly-GR was significantly higher in 36R-expressing flies vs. −RU control (**P = 0.0019, Welch’s two-tailed *t* test, +RU *n* = 4, −RU *n* = 3). No significant difference in poly-GR was observed in Sense-PolyA-1 (*P* = 0.4006, Welch’s two-tailed *t* test, +RU *n* = 4, −RU *n* = 3), Sense-Intronic-1 (*P* = 0.4226, Welch’s two-tailed *t* test, both conditions *n* = 3) or Sense-800 PolyA (*P* = 0.4226, Welch’s two-tailed *t* test, +RU *n* = 4, −RU *n* = 3). Bars are Mean ± SEM, individual replicates are shown as circles. Genotypes: w; UAS-Sense-PolyA-1/+; elavGS/+ (Sense-PolyA-1), w; UAS-Sense-Intronic-1/+; elavGS/+ (Sense-Intronic-1), w; UAS-Sense-800 PolyA/+; elavGS/+ (Sense-800 PolyA), w; UAS-Sense-1000 PolyA/+; elavGS/+ (Sense-1000 PolyA), w; UAS-Sense > 1000 PolyA/+; elavGS/+ (Sense > 1000 PolyA), w; UAS-AS-PolyA-1/+; elavGS/+ (AS-PolyA-1), w; UAS-AS-Intronic-1/+; elavGS/+ (AS-Intronic-1), w; UAS-36R/+; elavGS/+ (36R)

We first assessed all fly lines, sense and antisense, for expression of poly-GP, the only DPR to be produced from RAN translation of both sense and antisense RNA. We have previously observed robust expression of poly-GP in flies expressing 36 GGGGCC repeats (36R) using western blotting [37], and, as expected, we observed a large poly-GP signal in these flies compared to uninduced controls by MSD immunoassay (Fig. 5a). We additionally observed a low-level production of poly-GP, greater than in controls, in some of the sense- and antisense repeat-expressing lines, despite the presence of stop codons. This is potentially due to a limited amount of RAN translation occurring between the stop codons of the interrupted repeats. Interestingly, a stronger signal was observed in Sense-PolyA-1 flies compared to controls than in intronic or 1000 repeat-expressing lines. Sense-PolyA-1 flies probably express higher levels of poly-GP because the relatively short repeat length and nuclear export to the cytoplasm favour RAN translation in this line. Crucially, when we assessed the levels of the toxic DPR poly-GR, in sense RO repeat expressing lines that displayed a significant poly-GP signal, we failed to observe any poly-GR above background (Fig. 5b), whereas poly-GR was present in the brains of 36R expressing positive controls, as expected. This lack of poly-GR is consistent with the lack of toxicity observed in these lines.

## Discussion

Here, we have demonstrated that expression in adult neurons of both sense and antisense *C9orf72* mutation-associated repeat RNA does not produce typical neurodegeneration-associated phenotypes in *Drosophila,* despite the formation of sense and antisense cytoplasmic RNA, or nuclear RNA foci.

We, and others, have previously demonstrated that overexpression of *C9orf72* repeat RNA is extremely toxic to *Drosophila* neurons, resulting in a dramatically reduced lifespan [19, 37, 56]. However, the introduction of regularly interspersed stop codons, and the resulting reduced production of DPRs, prevents this toxicity, suggesting that it is dependent on DPR production in these flies [37], a finding further supported by our current study.

We have here demonstrated that the genomic context of repeat RNA (intronic vs. polyA) affects intranuclear RNA foci formation, presumably because splicing of the intronic repeats prevents the nuclear export of repeat RNA. This is in line with previous studies that have confirmed that intronic localisation of repeat RNA favours its nuclear retention and nuclear RNA foci formation [53, 54]. It has additionally been demonstrated that reduced nuclear export of repeat RNA via knockdown of SRSF1 enhances nuclear RNA foci formation in patient iPSC-derived astrocytes [24]. We have additionally observed enhanced foci formation with increasing repeat length, consistent with our previous observations in transfected cells [37]. It is interesting to note that although we unexpectedly detected RAN translated poly-GP in some of our stop codon-interrupted repeat lines, the highest levels of poly-GP protein were observed in Sense-PolyA-1 flies expressing ~ 100 repeats. In these flies repeat RNA was largely cytoplasmic, compared with intronic or 800 to > 1000 repeat expressing lines where RNA was predominantly accumulated in the nucleus. Interventions that affect nucleocytoplasmic transport have previously been demonstrated as modifiers of toxicity in *C9orf72* models [6, 19, 27, 58]. Interestingly, it has been shown that knockdown or chemical inhibition of the *Drosophila* ortholog of XPO1 (emb) significantly rescues toxicity in repeat-expressing flies, but enhances toxicity in recodonised DPR-expressing flies implicating XPO1 in the nuclear export of repeat GGGGCC RNA, and suggesting that preventing nuclear export of repeat RNA may be of therapeutic value [58].

The role of repeat RNA in the pathogenesis of *C9orf72* mutation-associated ALS/FTD is currently contentious. *C9orf72* repeat RNA forms secondary structures such as hairpins and G-quadruplexes, leading to the formation of intranuclear RNA foci, which are generally assumed to become toxic by the sequestration of RNA-binding proteins [23]. One study has found a correlation between the presence of antisense RNA foci and nuclear clearance of TDP-43 [13]; however, other studies have demonstrated that neither sense nor antisense nuclear RNA foci burden consistently correlate well with the region-specific neurodegeneration observed in patient post-mortem tissue [15, 38]. Additionally, despite the formation of large numbers of nuclear RNA foci in recent bacterial artificial chromosome (BAC) mouse models, toxicity is only observed in some of the mice [26, 34, 42, 44]. In one BAC mouse model regional neurodegenerative phenotypes were positively correlated with the presence of antisense RNA foci. However, the same study found that TDP-43 pathology and neurodegeneration could be observed independent of the formation of nuclear RNA foci in mice expressing short repeat lengths [34].

It is possible that hexanucleotide RNA is toxic through mechanisms independent of intranuclear RNA foci formation. Recently, it was demonstrated that toxicity correlates with the presence of neuritic RNA foci in sense RNA expressing primary neurons [9]. Our current models provide evidence that in *Drosophila* neither cytoplasmic nor nuclear RNA are sufficient to induce a strong pathological phenotype. This is consistent with the results of a previous study examining intronic sense foci in *Drosophila,* which also did not observe a neurodegenerative phenotype [53].

Previous studies have observed toxicity in *Drosophila* expressing (GGGGCC)_15_–CTCGAG–(GGGGCC)_15_ repeats [56, 58] or (GGGGCC)_48-58_ repeats [9, 19]. However, it is not clear whether toxicity is primarily driven through the presence of repeat RNA or DPRs. Here we have used newly described sensitive ELISA assays to assess accurately the level of poly-GP and poly-GR in our *Drosophila* models, without having to employ the strong overexpression paradigm necessary to detect DPRs by immunoblot [37, 58]. We note with interest, that in our model system soluble poly-GP was present at approximately 10 times the abundance of poly-GR, potentially due to a high rate of turnover of poly-GR [30], or the influence of as-yet-unknown cis factors in the RNA sequence. The lack of toxicity we observed in our RNA-only models is correlated with a lack of expression of poly-GR, consistent with its role in mediating the neurotoxic phenotypes seen in *Drosophila.* Notably, the only consistent toxic phenotype we observed in any genotype was a modest reduction in climbing ability in ~ 100 repeat Sense-PolyA flies as well as a slight rough-eye phenotype when these flies were crossed to GMR-Gal4. These flies showed the largest production of poly-GP protein, suggesting that DPRs present at low levels, or below the level of detection may still be capable of inducing mildly toxic phenotypes.

It is possible that proteins that bind to hexanucleotide RNA in humans are not well conserved in *Drosophila,* rendering flies resistant to RNA toxicity. Some identified sense RNA-binding proteins like Zfp106 do not have highly homologous orthologs in *Drosophila* [11]. However, generally RNA-binding proteins are well conserved between *Drosophila* and humans [21]. We observe sequestration of Glo, the *Drosophila* ortholog of hnRNP H, a commonly identified hexanucleotide RNA-interacting protein [12, 14, 22, 25, 32], suggesting that repeat RNA sequesters RNA-binding proteins in vivo in *Drosophila.* We have confirmed that Glo knockdown driven by RNAi is insufficient to induce rough-eye and eclosion phenotypes, consistent with a previous report which demonstrated a lack of eye phenotype and only a very slight defect in climbing ability when Glo knockdown is driven throughout development using the constitutively active elav-Gal4 driver [1]. These results, therefore, imply that although repeat RNA is capable of sequestering *Drosophila* RNA-binding proteins per se, that the particular RNA-binding proteins that are sequestered do not produce overt phenotypes associated with neurodegeneration in *Drosophila*.

Although we did not observe a typical neurodegenerative phenotype, a lifespan extension was observed in females in almost all repeat RNA expressing lines, and does not occur in controls. This lifespan extension does not correlate with the production of DPRs, suggesting that it is dependent on sense and antisense RNA. The mechanism causing this effect is currently unknown, but both sense and antisense *C9orf72*-associated hexanucleotide hairpins have been demonstrated to bind a large number of proteins with a similar degree of avidity in vitro, making a shared mechanism of action possible [22]. Additionally, it has previously been noted that repeat expansion disease-associated hairpin RNAs can induce similar transcriptional changes in *Drosophila* neurons despite having differing primary sequences [18]. Intriguingly, expression of hairpin RNA has been shown to disrupt the Akt/GSK3-β signalling pathway [18], the disruption of which we have previously identified as having pro-longevity effects in *Drosophila* [10]. Further work will be required to fully elucidate the mechanism(s) by which this occurs, but it does indicate some biological effect of sense and antisense repeat RNA, albeit not sufficient to cause overt toxicity.

A full understanding of the mechanisms by which the *C9orf72* repeat expansion leads to toxicity are essential in designing effective therapeutic interventions. Several groups have focused their efforts on antisense oligonucleotides (ASOs) targeting the sense RNA stand [17, 31, 47], or G-quadruplex binding molecules, which target the sense-strand-specific G-quadruplex RNA secondary structure [48, 57, 58]. These approaches prevent the formation of sense RNA foci and the production of the more highly abundant sense dipeptide proteins. However, if antisense RNA induces toxicity in patients, these interventions may be of limited clinical effectiveness. Our results suggest that sense and antisense RNA are well tolerated in *Drosophila*, suggesting that therapeutic interventions that act to reduce dipeptide repeat protein production will be of importance as a therapeutic endpoint.

## Electronic supplementary material

Below is the link to the electronic supplementary material.

Supplementary material 1 (PDF 13299 kb)
