# Supplementary material for: Sense and antisense RNA are not toxic in Drosophila models of C9orf72-associated ALS/FTD
Source: Acta Neuropathol. 2018 Jan 29;135(3):445–57. doi: 10.1007/s00401-017-1798-3 (PMC6385858; doi:10.1007/s00401-017-1798-3)
Supplement: Supplementary file 1 — Supplementary material 1 (PDF 13299 kb) [file 401_2017_1798_MOESM1_ESM.pdf]

<u>Electronic Supplementary Material</u>

Sense and antisense RNA are not toxic in *Drosophila* models of *C9orf72*-associated ALS/FTD

Thomas G. Moens[1,2], Sarah Mizielinska[1,3,4], Teresa Niccoli[1,2], Jamie S. Mitchell[1], Annora Thoeng[1], Charlotte E. Ridler[1], Sebastian Grönke[5], Jacqueline Esser[5], Amanda Heslegrave[6,8], Henrik Zetterberg[6,7,8], Linda Partridge[2,5]*, Adrian M. Isaacs[1,8]*

1. Department of Neurodegenerative Disease, UCL Institute of Neurology, London WC1N 3BG, UK.
2. Department of Genetics, Evolution and Environment, Institute of Healthy Ageing, University College London, London WC1E 6BT, UK.
3. Department of Basic and Clinical Neuroscience, Maurice Wohl Clinical Neuroscience Institute, Institute of Psychiatry, Psychology and Neuroscience, King's College London, London SE5 9RT, UK
4. UK Dementia Research Institute at King's College London, Maurice Wohl Clinical Neuroscience Institute, London SE5 9RT, UK
5. Max Planck Institute for Biology of Ageing, 50931 Cologne, Germany
6. Department of Molecular Neuroscience, UCL Institute of Neurology, London, WC1N 1PJ, UK.
7. Clinical Neurochemistry Laboratory, Institute of Neuroscience and Physiology, Department of Psychiatry and Neurochemistry, The Sahlgrenska Academy, University of Gothenburg, Sweden.
8. UK Dementia Research Institute at UCL, UCL Institute of Neurology, London WC1N 3BG, UK.

*To whom correspondence should be addressed:
Adrian Isaacs: a.isaacs@ucl.ac.uk, Tel: +44(0)20 7837 5470, Fax: +44(0)20 7837 8047
Linda Partridge: l.partridge@ucl.ac.uk, Tel: +44 (0)20 7679 4380

Materials and Methods

Southern blotting

DNA from approximately 30 female *Drosophila* was extracted using the Qiagen DNeasy Blood and Tissue Kit following manufacturer's instructions, before being incubated with 20μg/ml RNase A (Qiagen) for 1 hour at 37ºC. 40μg of genomic DNA was digested overnight at 37ºC with 20 units of DdeI and AluI (NEB). ~1152RO samples were run on a 0.8% agarose gel at 60V overnight, whilst ~100RO samples were run on a 1% gel for 6 hours. The gel was agitated for 30 minutes in 1L of denaturation buffer (National Diagnostics) followed by 1L of neutralisation buffer (National Diagnostics). DNA was transferred onto a positively charged nylon membrane (Roche Applied Science) via capillary action overnight using 20X SSC (saline sodium citrate, Fisher) as transfer buffer. Following transfer, DNA was baked onto the nylon membrane at 80ºC for 2 hours. Membrane was briefly wetted in ddH2O before being incubated in DIG easy Hyb buffer (Roche) with 100μg/ml salmon sperm DNA (Thermo) at 48ºC for 4 hours. It was then transferred into fresh Dig easy hyb buffer with salmon sperm and 1μg/ml of 5'-DIG-$(GGGGCC)_5$-DIG-3' probe (produced by Eurofins genomics) overnight at 48ºC. The blot was washed in 2xSSC with 0.1% SDS (sodium doecyl sulphate) while ramping in temperature from 48ºC-65ºC, followed by pre-warmed 0.5xSSC with 0.1% SDS at 65ºC for 15 minutes, and then 0.2xSSC w/ 0.1% SDS for 15 minutes at 65ºC. Following this, the blot was processed using the DIG wash and block buffer set (Roche) following manufacturer's instructions. The membrane was incubated in CDPstar ready to use (Roche) before being exposed to film (Roche Lumi-Film chemiluminescent detection film) for >1 hour before being developed using an X4 automatic processor (XOgraph). Due to technical issues with probe synthesis, some blots were incubated with 5'-DIG-$(CCCCGG)_5$-DIG-3' (antisense) probe.

Eye GFP fluorescence

Flies were reared at 25°C. Five days after eclosion heads were mounted on glass slides and imaged using a Zeiss Axioskop 2 plus microscope using the same settings for each image.

Eye phenotyping and eclosion experiments

3 GMR-Gal4 virgins were crossed to males of the indicated genotypes on SYA food, 5-6 vials per genotype were set up. Females were allowed to lay for 24 hours and eggs left at 29°C to hatch and eclose. Eye phenotypes were scored 24 hours after eclosion, based on a 5-point grading scale: 1= no rough eye, 2= slight rough eye, 3= moderate rough eye, 4=severe rough eye, 5= very severe / no eye. A minimum of 3-5 eyes were scored per sex per vial (n=21-30 per sex, per genotype). The strong eclosion effect in some vials meant that for GR36 flies less flies were scored (n=10-28 per sex, per experiment). Representative images of eyes were taken 24-48hr after eclosion using a Leica M165 C microscope by taking serial images at different depths and reconstructing the final image using the Leica application suite software. 72 hours after eclosion, the number of pupal cases and the number of adult flies were counted and used to calculate the % of adult flies that had eclosed successfully. Experimenter was blind to genotype when scoring both assays.

Immunoblotting

24hr after eclosion the retinas of female flies were removed in ice cold PBS, 10 retinas per replicate were transferred to 40µl of 2x laemmli sample buffer (BioRad) with 100mM DTT. Tissue was homogenised before being boiled at 95°C for 10 minutes. 20µl of sample was loaded per well in a 10 well 4-12% Novex Bis-Tris gel (Invitrogen). Protein was transferred to a nitrocellulose membrane using the Trans-Blot Turbo transfer system (BioRad) and blocked in 2% skimmed milk in TBS for 1 hour at room temperature. Anti-Glorund (5B9, DSHB) was applied at 1/750 concentration in block overnight at 4°C. The blot was washed in TBS-(0.1%) Tween and incubated in anti-mouse-HRP (Abcam, ab6789) at 1/10,000 in block. Glorund was visualised using SuperSignal West Femto Maximum Sensitivity Substrate (Thermo). Following this, anti β-actin (Abcam, ab8224) was applied for 1 hour at room temperature at 1/10,000 in TBS-tween with 5% skimmed milk. Following washes, the blot was incubated with anti-mouse-HRP (Abcam, ab6789) at 1/10,000 in block. Actin was visualised using Luminata

Forte Western substrate (Millipore). Bands were normalised relative to β-actin using ImageJ software.

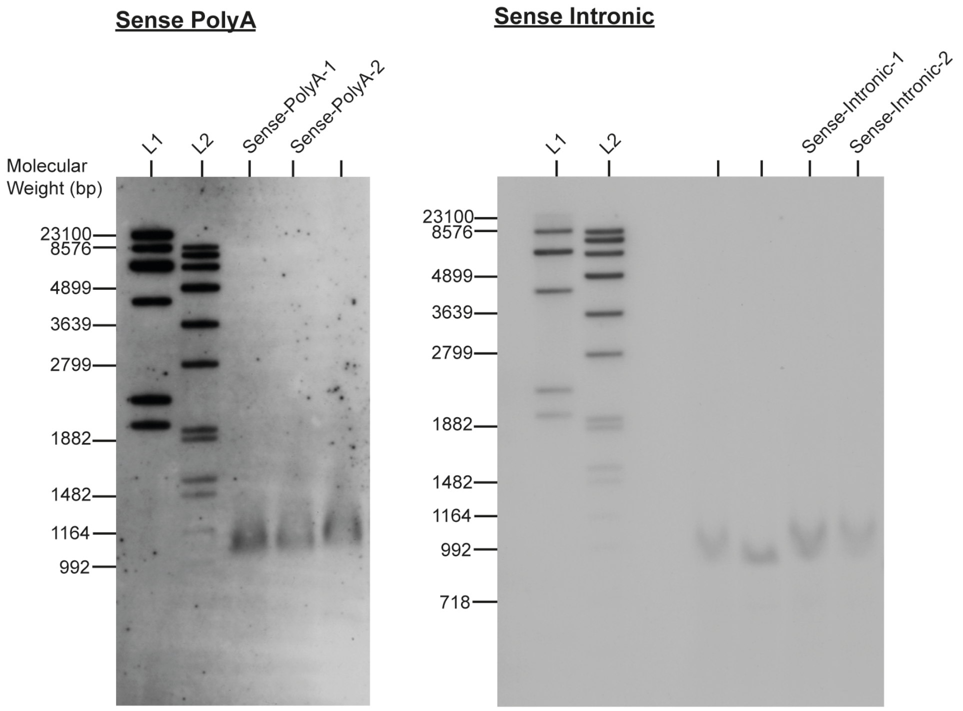

Fig. 1 Southern blots confirm insertion size in transgenic ~100 sense repeat *Drosophila.* Southern blots were performed using genomic DNA derived from multiple independent transgenic lines (indicated by tick marks). Insertions were sized by comparison to two DNA ladders (L1 and L2, molecular weight shown in base pairs). Two independent lines were selected for each type of construct based on repeat size: Sense-PolyA-1 and Sense-PolyA-2 (expected band size=982bp), and Sense-Intronic-1 and Sense-Intronic-2 (expected band size= 825bp)

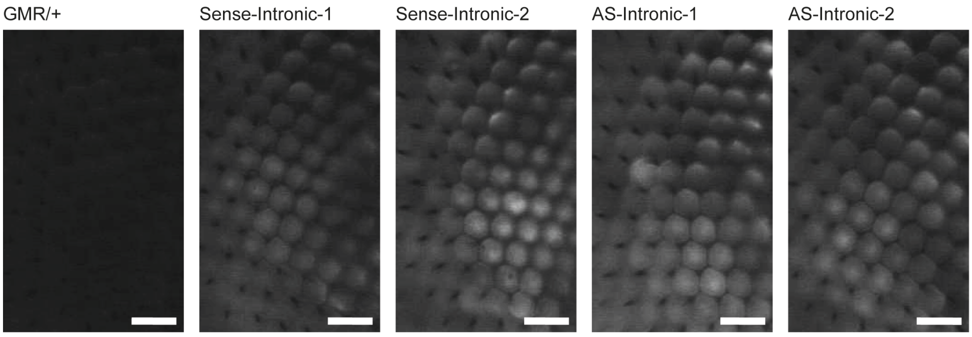

Fig. 2 Confirmation of eGFP expression in intronic flies. Images of *Drosophila* eyes expressing constructs using the GMR-Gal4 driver. eGFP expression is observable in all intronic lines, but not in the heterozygous driver alone (GMR/+) condition. Scale bar 25µm.
Genotypes: w; GMR-Gal4/+ (GMR/+), w; GMR-Gal4/UAS-Sense-Intronic-1 (Sense-Intronic-1), w; GMR-Gal4/UAS-Sense-Intronic-2 (Sense-Intronic-2), w; GMR-Gal4/UAS-AS-Intronic-1 (AS-Intronic-1), w; GMR-Gal4/UAS-AS-Intronic-2 (AS-Intronic-2).

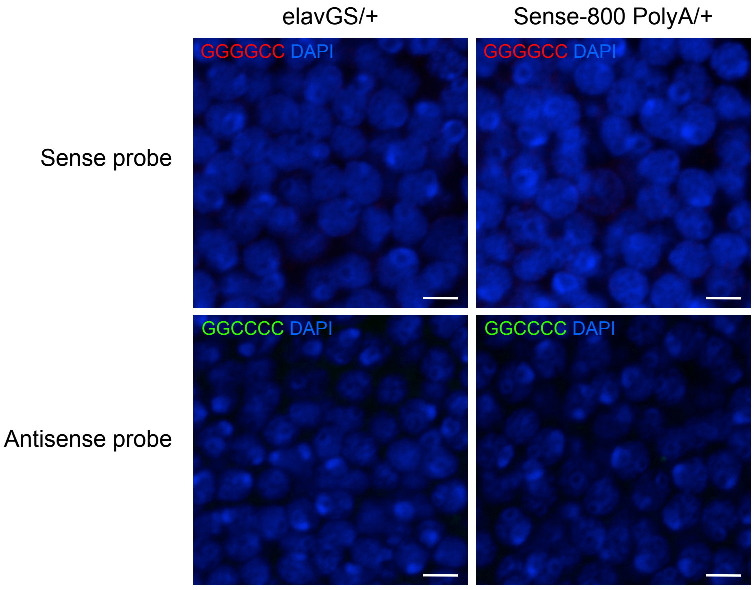

Fig. 3 Images of RNA FISH in control genotypes. RNA foci-like structures are not observed in neurons in the driver-only control (elavGS/+) using either probes to detect sense or antisense hexanucleotide RNA. RNA-foci-like structures are rarely observed in flies heterozygous for hexanucleotide transgenes (Sense-800 PolyA/+). Scale bar 2.5µm. Genotypes: w; +; elavGS/+ (elavGS/+) and w; UAS-Sense-800 PolyA/+; + (Sense-800 PolyA/+)

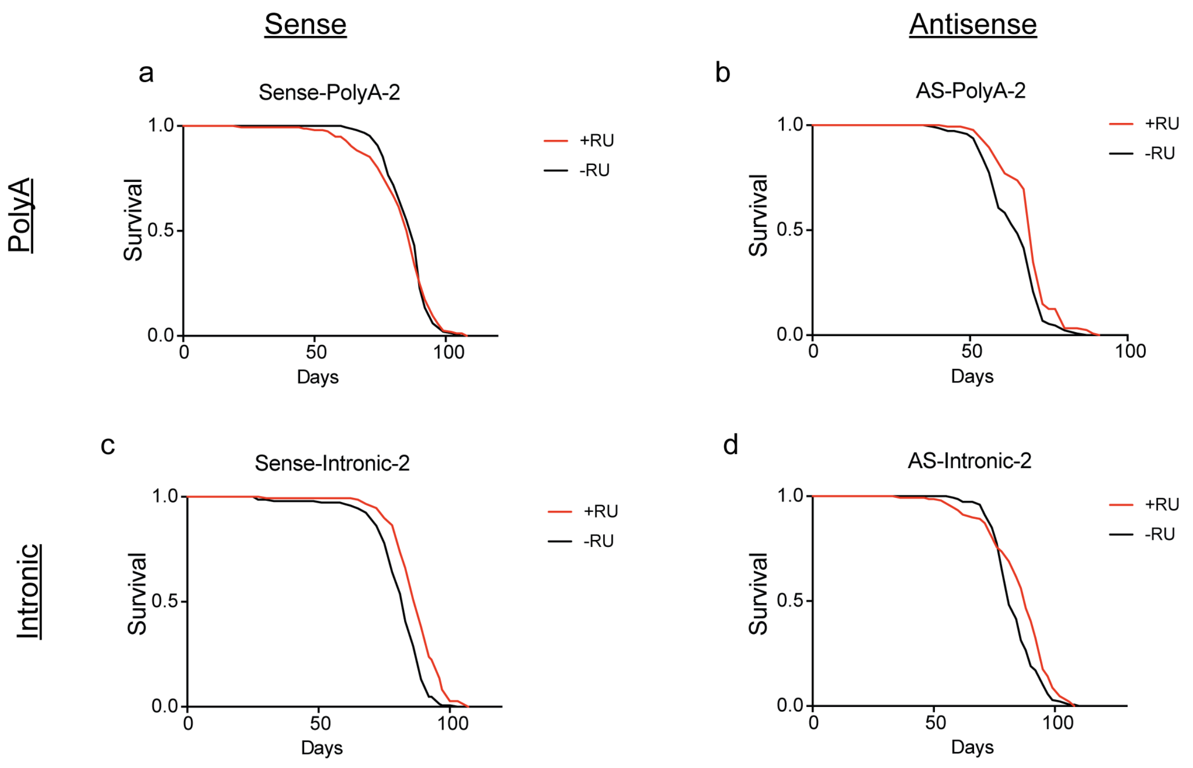

Fig. 4 Expression of sense and antisense RNA does not shorten lifespan in additional independent fly lines. Lifespan was assessed in the indicated lines **a** Sense-PolyA-2 expressing flies showed no difference in lifespan vs. controls (median lifespan –RU=86.5 +RU=86.5, P=0.84 log rank test). **b** AS-PolyA-2 expressing flies showed a significant extension of lifespan vs. controls (median lifespan –RU=64.0 +RU=68.5, P=3.63E-05 log rank test). **c** Sense-Intronic-2 expressing flies showed a significant extension of lifespan vs. controls (median lifespan –RU=82 days +RU=87.5 days, P=7.38E-09). **d** AS-Intronic-2 expressing flies showed a significant extension of lifespan vs. controls (median lifespan –RU=79.5 +RU=87, P=0.00117). Genotypes: w; UAS-Sense-PolyA-2/+; elavGS/+ (Sense-PolyA-2), w; UAS-Sense-Intronic-2/+; elavGS/+ (Sense-Intronic-2), w; UAS-AS-PolyA-2/+; elavGS/+ (AS-PolyA-2), w; UAS-AS-Intronic-2/+; elavGS/+ (AS-Intronic-2)

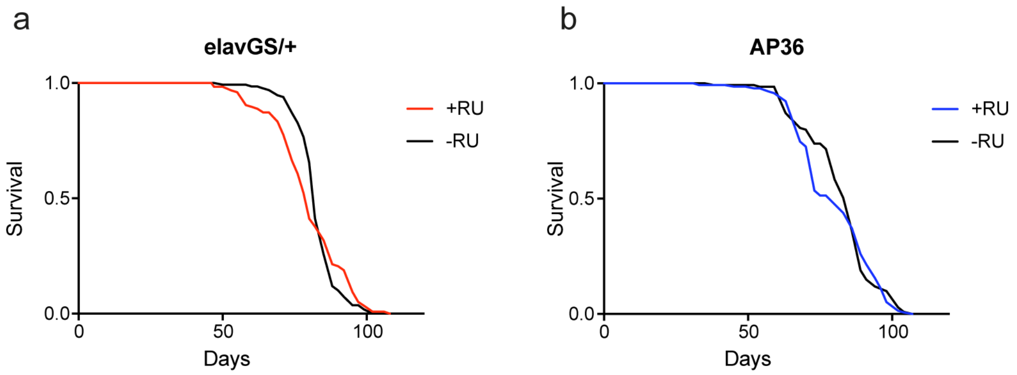

Fig. 5 No lifespan extension is observed from driver alone or non-toxic protein expression. **a** Lifespan extension is not observed in flies in response to feeding RU486 in flies carrying the driver alone (elavGS/+) (median lifespan –RU=81.0 days, +RU=79.0 days, P=0.63 log rank test). **b** Lifespan extension is not seen in flies expressing AP36 (median lifespan –RU=84.5 days, +RU=78.5 days, P=0.46 log rank test). Genotypes: w; +; elavGS/+ (elavGS/+), w; UAS-AP36/+; elavGS/+ (AP36)

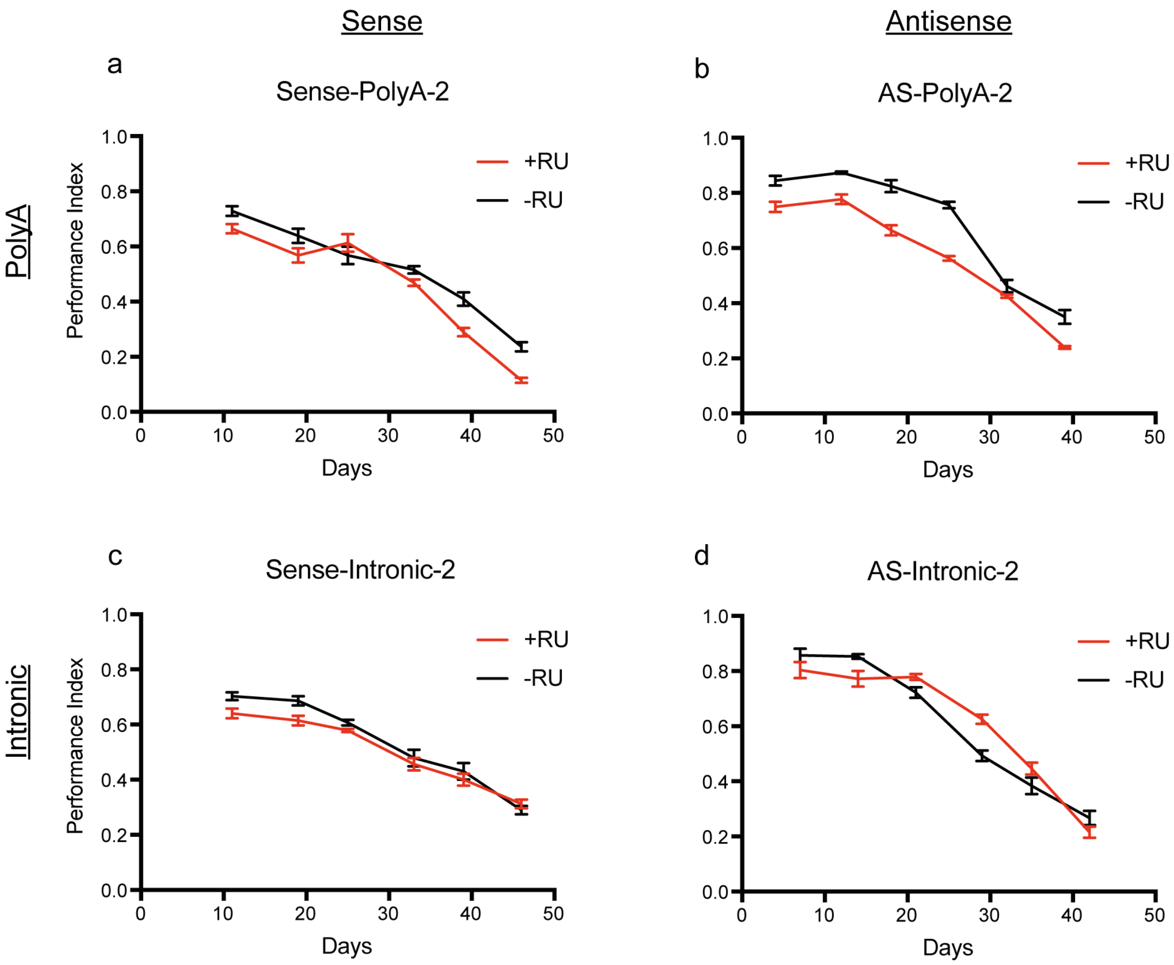

Fig. 6 Expression of sense and antisense RNA does not strongly affect climbing ability in additional independent fly lines**.** Climbing ability was analysed in the indicated lines. **a** Negative geotaxis assays performed on Sense-PolyA-2 flies expressing the transgene (+RU) and controls (-RU). A slight reduction in climbing ability with age is observed in Sense-PolyA-2 flies compared to controls (ordinal logistic regression, interaction of RU status and time P=0.049). **b** No significant difference in climbing ability is observed in AS-PolyA-2 expressing flies with age (ordinal logistic regression, interaction of RU status and time P=0.0618). **c** A slight reduction in climbing ability with age is observed in Sense-Intronic-2 flies (ordinal logistic regression, interaction of RU status and time P=0.0139). **d** A slight improvement of climbing ability with age is observed in AS-Intronic-2 flies (ordinal logistic regression, interaction of RU status and time P=0.0391). Genotypes: w; UAS-Sense-PolyA-2/+; elavGS/+ (Sense-PolyA-2), w; UAS-Sense-Intronic-2/+; elavGS/+ (Sense-Intronic-2), w; UAS-AS-PolyA-2/+; elavGS/+ (AS-PolyA-2), w; UAS-AS-Intronic-2/+; elavGS/+ (AS-Intronic-2)

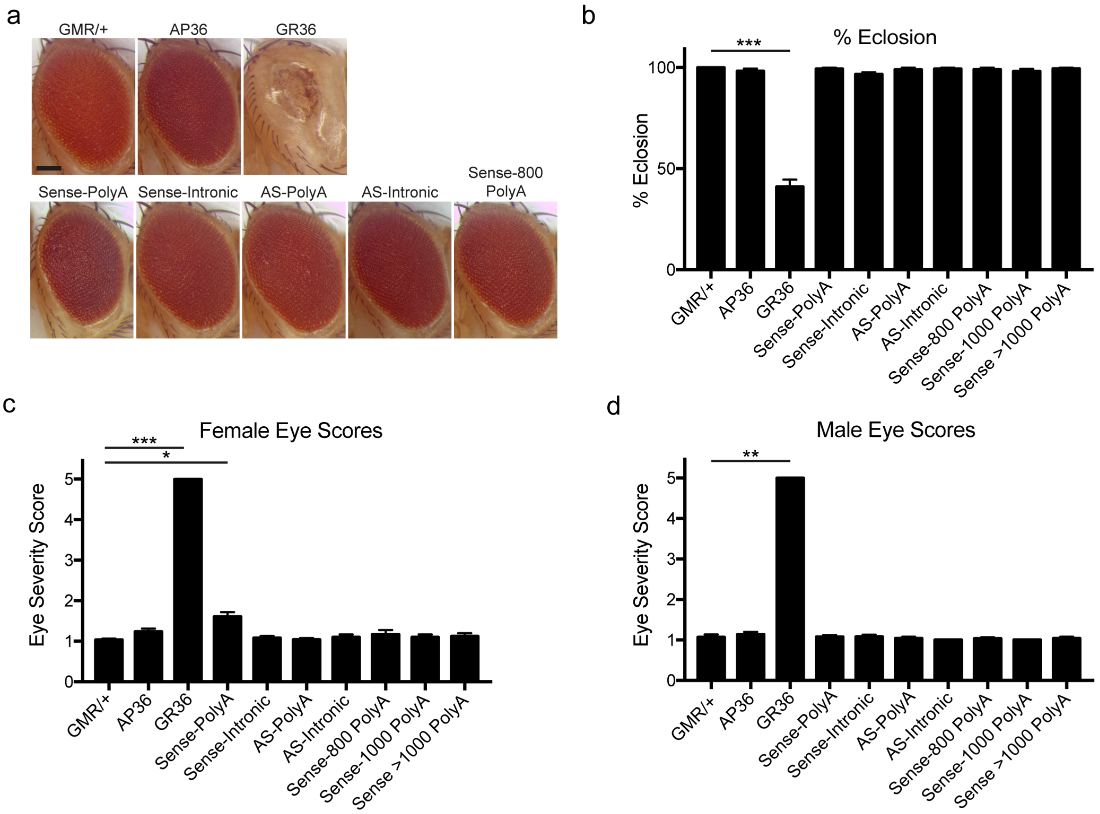

Fig. 7 Expression of *C9orf72* sense and antisense RNA-only repeats under the GMR-Gal4 driver is not strongly toxic to Drosophila at 29°C. Flies were crossed to the GMR-Gal4 driver to determine whether rough eye phenotypes or an eclosion defect was induced. **a** Representative images of eyes of female flies carrying the indicated insertion expressed under the GMR-Gal4 driver. GMR/+ is the heterozygous driver alone. UAS-GR36 and UAS-AP36 were used as previously validated positive and negative controls respectively [37]. Scale bar 100µm. **b** % of flies that eclosed from pupae for each line. A significant eclosion defect is observed in GR36 expressing flies compared to controls but no other lines (Kruskall-Wallis effect of genotype P=0.0009, post-hoc Dunn's multiple comparisons between GMR/+ and GR36, ***P=0.0003). Bars are mean % eclosion per vial ± SEM. **c** Eye severity scores for females of the individual lines tested. A significant rough eye phenotype was observed in GR36 expressing controls (Kruskall-Wallis test, effect of genotype P=0.0001, with post-hoc Dunn's multiple comparisons between GMR/+ and GR36, ***P=0.0004), whilst a slight rough eye phenotype was observed in Sense-PolyA expressing flies (post-hoc Dunn's multiple comparisons between GMR/+ and Sense-PolyA, *P=0.0139). Bars are mean eye score per vial ± SEM. **d** Eye severity scores for males of the individual lines tested. A significant rough eye was observed in GR36 expressing flies but not other lines (Kruskall-Wallis effect of genotype P=0.0006 with post-hoc Dunn's multiple comparisons between GMR/+ and GR36, **P=0.0016). Bars are mean eye score per vial ± SEM. Genotypes: w; GMR-Gal4/+ (GMR/+), w; UAS-AP36/ GMR-Gal4 (AP36), w; UAS-GR36/ GMR-Gal4 (GR36), w; UAS-Sense-PolyA-2/ GMR-Gal4 (Sense-PolyA), w; UAS-Sense-Intronic-2/ GMR-Gal4 (Sense-Intronic), w; UAS-AS-PolyA-2/ GMR-Gal4 (AS-PolyA), w; UAS-AS-Intronic-2/ GMR-Gal4 (AS-Intronic), w; UAS-Sense-800 PolyA/ GMR-Gal4 (Sense-800 PolyA), w; UAS-Sense-1000 PolyA/ GMR-Gal4 (Sense-1000 PolyA), w; UAS-Sense >1000 PolyA/ GMR-Gal4 (Sense >1000 PolyA).

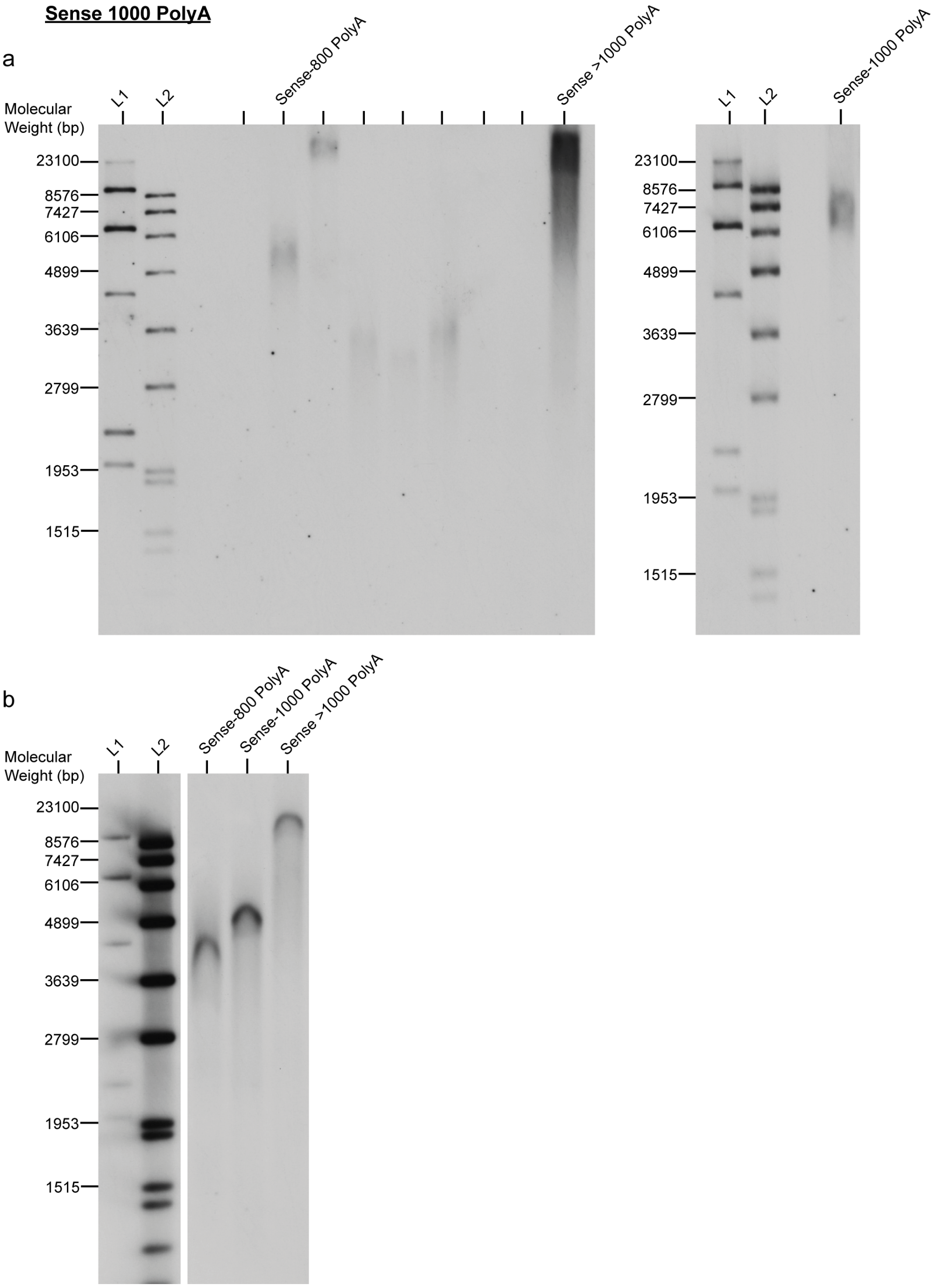

Fig. 8 Southern blots confirm insertion size in transgenic ~1000 repeat *Drosophila.* Southern blots were performed using genomic DNA derived from multiple independent transgenic lines (indicated by tick marks). Insertions were sized by comparison to two DNA ladders (L1 and L2, molecular weight shown in base pairs). **a** Two lines Sense-800-PolyA and Sense-1000-PolyA carry expansions approximately equivalent to expected size (~800 repeats, and ~1000 repeats respectively, expected band size=7768bp). Some lines were observed to reproducibly carry expansions larger than expected. One of these lines Sense>1000 PolyA was selected for further characterisation (estimated repeat length ~2000-5000). **b** Southern blot performed on DNA extracted from flies derived from the same cross used in climbing and negative geotaxis assays (genotypes as in Fig. 2). Results indicate that flies carry large expansions equivalent to ~650 repeats, ~800 repeats and >1000 repeats in lines Sense-800-PolyA, Sense-1000-PolyA and Sense>1000 PolyA respectively, demonstrating that repeat lengths had not substantially retracted.

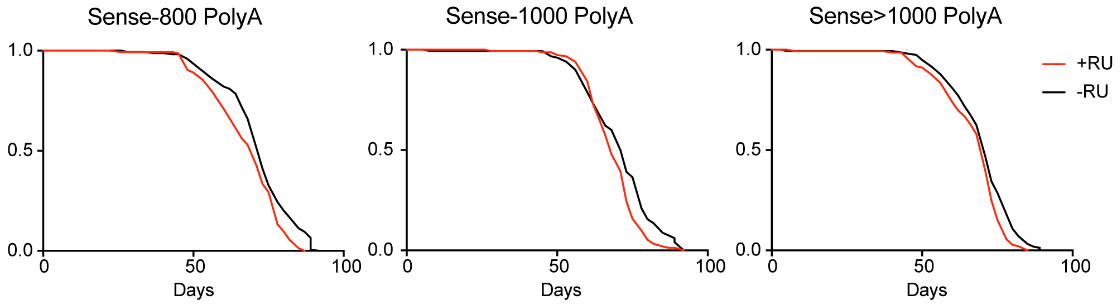

Fig. 9 Expression of sense 1000 RNA-only repeats modestly reduces lifespan in male flies. A modest reduction in lifespan was seen across all lines. Sense-800 PolyA (median lifespan –RU=72 days +RU=69.5 days, P=0.00073 log rank test), Sense-1000 PolyA (median lifespan –RU=69.5 days +RU=67 days, P=0.0018 log rank test), Sense>1000 PolyA (median lifespan –RU=69.5 +RU=69.5, P=0.003 log rank test). Genotypes: w; UAS-Sense-800 PolyA/+; elavGS/+ (Sense-800 PolyA), w; UAS-Sense-1000 PolyA/+; elavGS/+ (Sense-1000 PolyA), w; UAS-Sense>1000 PolyA/+; elavGS/+ (Sense>1000 PolyA)

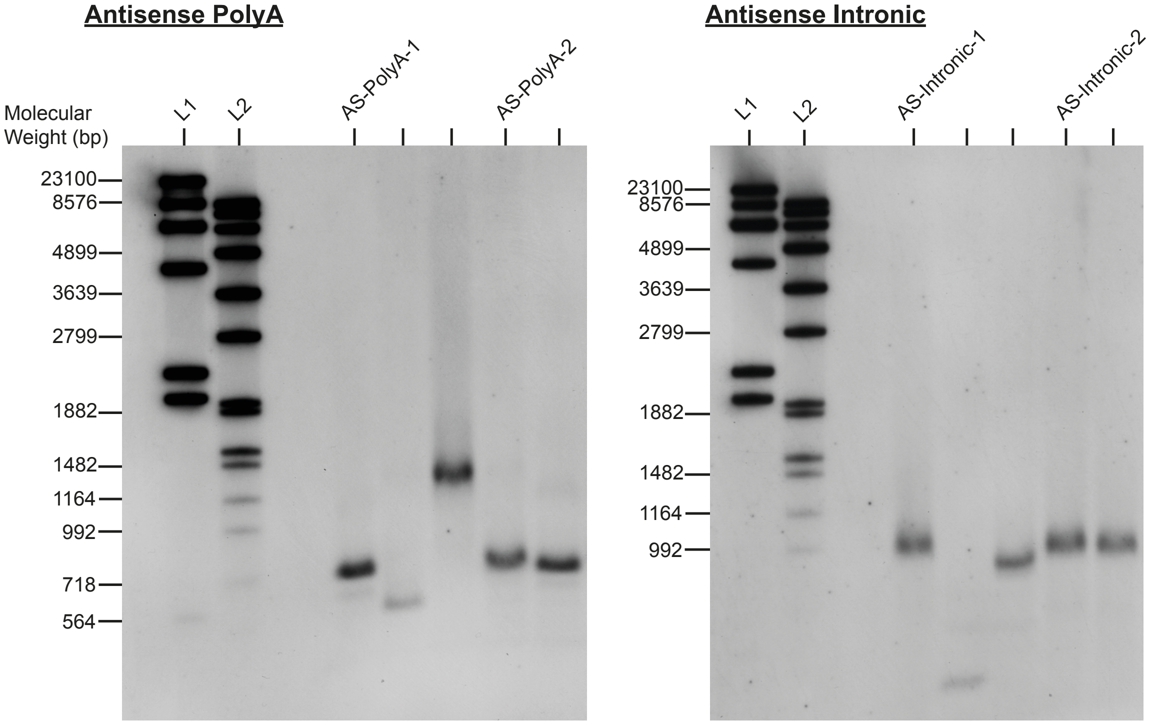

Fig. 10 Southern blots confirm insertion size in antisense transgenic *Drosophila.* Southern blots were performed using genomic DNA derived from multiple independent transgenic lines (indicated by tick marks). Insertions were sized by comparison to two DNA ladders (L1 and L2, molecular weight shown in base pairs). Two independent lines were selected for each type of construct based on repeat size: AS-PolyA-1 and AS-PolyA-2 (expected band size=956bp), and AS-Intronic-1 and AS-Intronic-2 (expected band size= 865bp)

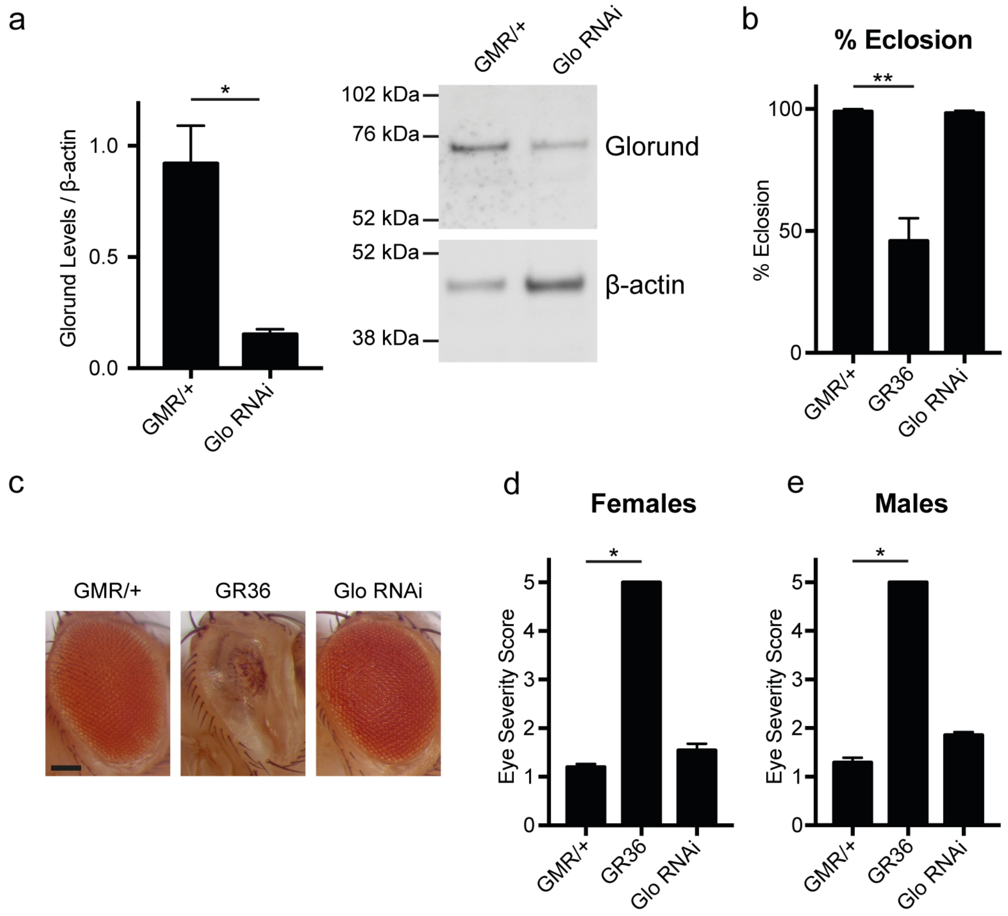

Fig. 11 Partial Glorund loss of function does not induce toxicity **a** An RNAi line against Glorund was crossed to the GMR-Gal4 driver and adult retinas subjected to immunoblotting to confirm knockdown of Glorund protein relative to the heterozygous driver alone (GMR/+). Representative blot, and graph depicting relative protein expression are shown. Glorund expression was significantly reduced by RNAi compared to controls (two-tailed Welch's t test, *P=0.0434). Bars are mean ±SEM (n=3 replicates per condition) **b** Graph depicting % of flies that eclosed from pupae for each line. A significant eclosion defect is observed in GR36 expressing positive control flies compared to controls but no other lines (Kruskall-Wallis effect of genotype P=0.0012, post-hoc Dunn's multiple comparisons between GMR/+ and GR36, **P=0.0073). Bars are mean % eclosion per vial ± SEM. **c** Representative images of eyes of female flies carrying the indicated insertion expressed under the GMR-Gal4 driver. GMR/+ is the heterozygous driver alone. UAS-GR36 was used as a positive control. Scale bar 100µm. **d** Eye severity scores for females of the indicated lines. A significant rough eye was observed in GR36 expressing flies but not other lines (Kruskall-Wallis test, effect of genotype P=0.0002, with post-hoc Dunn's multiple comparisons between GMR/+ and other lines, *P=0.0020). Bars are mean eye score per vial ± SEM. **e** Eye severity scores for males of the indicated lines. A significant rough eye was observed in GR36 expressing flies but not other lines (Kruskall-Wallis test, effect of genotype P<0.0001, with post-hoc Dunn's multiple comparisons between GMR/+ and other lines, *P=0.0028). Bars are mean eye score per vial ±SEM. Genotypes: w; GMR-Gal4/+ (GMR/+), w; UAS-GR36/ GMR-Gal4 (GR36), w; GMR-Gal4/+; UAS-Glo RNAi/+ (Glo RNAi).

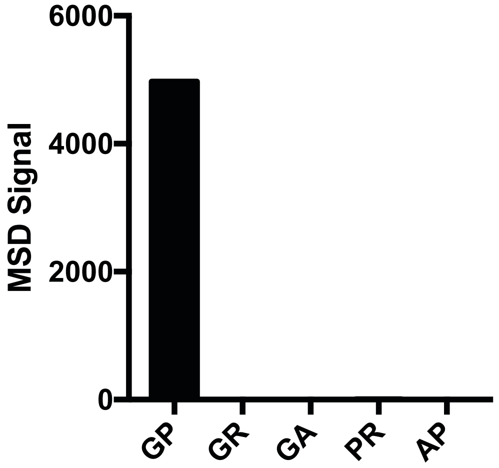

Fig. 12 Validation of poly-GP antibody. GP antibody was assessed against synthetic peptides demonstrating that the response is specific to poly-GP. The specificity of the GR antibody was assessed elsewhere [49].

Comparison			P value
Sense-800 PolyA/+ , elavGS/+	vs.	Sense-PolyA-1, Sense-PolyA-2, Sense-Intronic-1, Sense-Intronic-2, Sense 800-PolyA, Sense 1000-PolyA, Sense>1000 PolyA	3.10E-07
Sense-PolyA-1, Sense-PolyA-2	vs.	Sense-Intronic-1, Sense-Intronic-2	0.0071
Sense-PolyA-1, Sense-PolyA-2, Sense-Intronic-1, Sense-Intronic-2	vs.	Sense 800-PolyA, Sense 1000-PolyA, Sense>1000 PolyA	9.00E-09
Sense 800-PolyA, Sense 1000-PolyA	vs.	Sense>1000 PolyA	0.0039
Sense-PolyA-1	vs.	Sense-PolyA-2	0.9371
Sense-Intronic-1	vs.	Sense-Intronic-2	0.2344
Sense-800 PolyA	vs.	Sense-1000 PolyA	0.5608
elavGS/+	vs.	Sense-800 PolyA/+	0.953

Table 1: Summary of the orthogonal contrasts performed on the data in Fig. 2b

Comparison			P value
Sense-800 PolyA/+ , elavGS/+	vs.	AS-PolyA-1, AS-PolyA-2, AS-Intronic-1, AS-Intronic-2	2.60E-05
AS-PolyA-1, AS-PolyA-2	vs.	AS-Intronic-1, AS-Intronic-2	4.60E-05
AS-Intronic-1	vs.	AS-Intronic-2	0.0599
AS-PolyA-1	vs.	AS-PolyA-2	0.4143
elavGS/+	vs.	Sense-800 PolyA/+	0.9182

Table 2: Summary of the orthogonal contrasts performed on the data in Fig. 3b
